# Supplementary material for: End Sequence Analysis Toolkit (ESAT) expands the extractable information from single-cell RNA-seq data
Source: Genome Res. 2016 Oct;26(10):1397–410. doi: 10.1101/gr.207902.116 (PMC5052061; doi:10.1101/gr.207902.116)
Supplement: Supplemental Material [file supp_gr.207902.116_Supp_Materials.tar.gz › ESAT-esat_v0.1/doc/html/classumms_1_1core_1_1alignment_1_1_feature_sequence_alignment-members.html]

ESAT: Member List


ESAT


- Main Page
- Related Pages
- Packages
- Classes
- Files

- Class List
- Class Index
- Class Hierarchy
- Class Members

All Classes Namespaces Files Functions Variables Enumerator Pages

- umms
- core
- alignment
- FeatureSequenceAlignment

umms.core.alignment.FeatureSequenceAlignment Member List

This is the complete list of members for umms.core.alignment.FeatureSequenceAlignment, including all inherited members.

|  |  |  |
| --- | --- | --- |
| DEFAULT\_GAP\_EXTEND\_PENALTY | umms.core.alignment.FeatureSequenceAlignment | static |
| DEFAULT\_GAP\_OPEN\_PENALTY | umms.core.alignment.FeatureSequenceAlignment | static |
| DEFAULT\_MATCH\_SCORE | umms.core.alignment.FeatureSequenceAlignment | static |
| DEFAULT\_MISMATCH\_SCORE | umms.core.alignment.FeatureSequenceAlignment | static |
| FeatureSequenceAlignment(String featureBedFile, String genomeFasta) | umms.core.alignment.FeatureSequenceAlignment |  |
| FeatureSequenceAlignment(Collection< Gene > genes, String genomeFasta) | umms.core.alignment.FeatureSequenceAlignment |  |
| FeatureSequenceAlignment(String featureBedFile, String genomeFasta, float matchScore, float mismatchScore, float gapOpenPenalty, float gapExtendPenalty) | umms.core.alignment.FeatureSequenceAlignment |  |
| FeatureSequenceAlignment(Collection< Gene > genes, Map< String, broad.core.sequence.Sequence > chrs, float matchScore, float mismatchScore, float gapOpenPenalty, float gapExtendPenalty) | umms.core.alignment.FeatureSequenceAlignment |  |
| FeatureSequenceAlignment(Collection< Gene > genes, String genomeFasta, float matchScore, float mismatchScore, float gapOpenPenalty, float gapExtendPenalty) | umms.core.alignment.FeatureSequenceAlignment |  |
| getAllPairwiseAntisenseAlignments(float minAlignLength, float minPctIdentity) | umms.core.alignment.FeatureSequenceAlignment |  |
| getAllPairwiseSenseAlignments(float minAlignLength, float minPctIdentity) | umms.core.alignment.FeatureSequenceAlignment |  |
| main(String[] args) | umms.core.alignment.FeatureSequenceAlignment | static |
| writeAllAntisenseAlignmentsToBed(String outBedFile, int minAlignLength, float minPctIdentity) | umms.core.alignment.FeatureSequenceAlignment |  |
| writeAllAntisenseAlignmentsToBed(String outBedFile, boolean append, int minAlignLength, float minPctIdentity) | umms.core.alignment.FeatureSequenceAlignment |  |
| writeAllSenseAlignmentsToBed(String outBedFile, int minAlignLength, float minPctIdentity) | umms.core.alignment.FeatureSequenceAlignment |  |
| writeAllSenseAlignmentsToBed(String outBedFile, boolean append, int minAlignLength, float minPctIdentity) | umms.core.alignment.FeatureSequenceAlignment |  |


---

Generated on Fri Aug 15 2014 15:11:07 for ESAT by  

 1.8.7
